# Screening, isolation and molecular identification of biodegrading mycobacteria from Iranian ecosystems and analysis of their biodegradation activity

**DOI:** 10.1186/s13568-017-0472-4

**Published:** 2017-09-20

**Authors:** Davood Azadi, Hasan Shojaei, Sina Mobasherizadeh, Abass Daei Naser

**Affiliations:** 1 Department of Microbiology, School of Medicine, Isfahan University of Medical Sciences, Isfahan, Iran; 2 Infectious Diseases and Tropical Medicine Research Center, Isfahan University of Medical Sciences, Isfahan, Iran

**Keywords:** *Mycobacterium*, Bioremediation, 16S rRNA sequencing, Phylogeny

## Abstract

Anthropogenic origin pollutants including pesticides, heavy metals, pharmaceuticals and industry chemicals impose many risks to human health and environment and bioremediation has been considered the strategy of choice to reduce the risk of hazardous chemicals. In the current study, we aimed to screen and characterize mycobacteria from the diverse range of Iranian aquatic and terrestrial ecosystems with harsh and unfavorable environmental conditions that can be utilized for biodegradation of target pollutants. Mycobacteria were isolated from a collection of 90 environmental samples and identified to the species level using conventional microbiological and molecular methods including the PCR amplification of *hsp*65 and sequence analysis of, 16S rRNA genetic markers. The growth rate of the isolates in presence of pollutants, chromatography, Gibbs and turbidometric methods were used to assess their biodegradation activity. A total of 39 mycobacterial isolates (43.3%) were recovered from 90 samples that belonged to 21 various species consisting of *M. fortuitum*; 6 isolates, *M. flavescens* and *M. paragordonae*; 4 isolates each, *M. monacense, M. fredriksbergense and M. aurum;* 2 isolates each, 7 single isolates of *M. conceptionense, M. porcinum, M. simiae, M. celeriflavum, M. novocastrense, M. neoaurum, M. obuense* and 12 isolates that belonged to 8 unknown potentially novel mycobacterial species. The isolates were categorized in three groups based on their bioremediation activity, i.e., 5 (12.8%) organisms without biodegradation activity, 20 (51.2%) organisms with previously reported biodegradation activity, and 14 (35.9%) organisms that showed biodegradation activity but not previously reported. Our results showed that the Iranian ecosystems harbor a good reservoir of diverse mycobacterial species with biodegrading potentiality for neutralizing environmental chemical pollutants.

## Introduction

During past decades and with the advances in science and technology various chemicals and synthetic products such as petroleum derivatives like petrochemicals and plastic, insecticides and herbicides, radioactive substances and many other similar materials were increasingly introduced into the environment (Eisenbud and Gesell 1997; El-Shahawi et al. 2010; Petry et al. 1996; Samanta et al. 2002; White et al. 2010). Most of these contaminants are of anthropogenic origin and derived from industrial effluent discharges, point contaminant spills, and diffuse agricultural sources (Ritter et al. 2002; Schwarzenbach et al. 2010). Once in the environment, contaminants can be considered as a source for toxicity and pose adverse effect on the health of living organisms (Mastrangelo et al. 1996; Oliva et al. 2001; Reigart 2009; Thompson et al. 2009). In recent years, numerous chemical and physical methods such as dredging and incineration, evaporation, light oxidation, chemical oxidation, adsorption and leaching of soil particles have been applied to decompose and recycle these materials (Arias-Estévez et al. 2008; Perelo 2010; Schaer et al. 2001). However, these approaches are not cost effective and ecofriendly and may cause further damage to the environment by producing and emitting secondary pollutants and in some other cases dispersing pollutant agents make them much more bioavailable and increases their toxicity risk (Khan et al. 2004; Mulligan et al. 2001; Virkutyte et al. 2002). Finding an ecofriendly and rapid degradation approach for these hazardous materials could make a change in promoting public and environmental health. Bioremediation is one of these methods that has attracted much interest amongst scientists and environmentalists for degradation and neutralization of pollutants. Bioremediation is a process that uses organisms, mostly microorganisms and plants, to degrade, reduce toxicity or detoxify waste products and pollutants (Kumar et al. 2011). Therefore, the most important step in bioremediation process is the isolation and characterization of microorganisms capable of decontamination of a particular or a group of pollutants (Adams et al. 2015).

Numerous soil and aquatic bacterial species can breakdown and consume pollutants as a sole carbon and/or energy source, such as *Pseudomonas, Burkholderia, Bacillus, Polaromonas, Sphingomonas* genera and members of actinomycetes such as *Mycobacterium, Rhodococcus* and *Nocardia* (Leja and Lewandowicz 2010; Megharaj et al. 2011; Ward and Singh 2014). Actinomycetes which are among the most abundant groups of microorganisms in soil and water have been considered as a suitable candidate for implementation in bioremediation process due to their high catabolic capacity as well as survival and persistence in unfavorable environmental conditions. Actinomycetes are one of the dominant bacterial phyla on earth, a group of terrestrial or aquatic gram-positive bacteria with very complex to very simple cell structure, capable of decomposing a high variety of organic materials, fixing nitrogen, and producing secondary metabolites (Sharma 2014; Watanabe 2001). Thus, in the current study, we aimed to screen, identify and characterize environmental nontuberculous mycobacteria (NTM) from Iranian ecosystems and to assess their bioremediation activity that can contribute in development and enhancement of bioremediation technology.

## Materials and methods

### Sampling

**Fig. 1:**
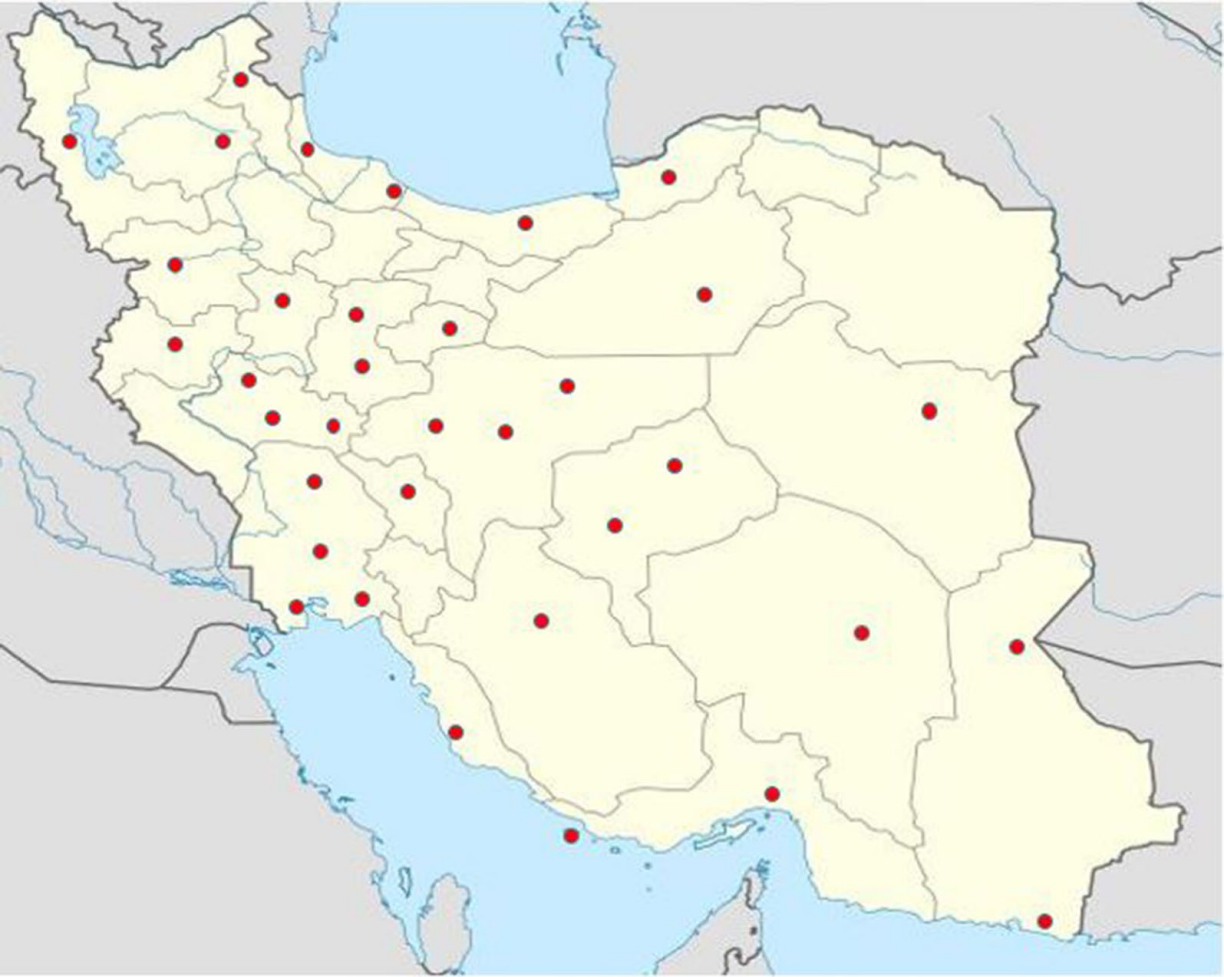
Geographic distribution of sampling site from Iran’s ecosystems

In a period of over 2 years between July 2014 and August 2016, a total number of 90 environmental samples, from ecologically diverse sources in Iran; i.e., drinking and non-drinking water, sea and river sediments, and effluents from municipal wastewater, hospitals, private households, industries, animal husbandries, were collected aseptically in sterile bottles and containers (Fig. 1; Table 1).

**Table 1:** Samples profile, phenotypic and molecular features and bioremediation analysis of mycobacterial isolates from Iranian ecosystems

Sample profile					Phenotypic features									16S rRNA analysis			Biodegradation activity
Isolates	Location (city) Province	Source	pH	Temperature	Opt. Tm	Growth rate	Semi quantitative catalase	Tween 80 hydrolyses	Tolerance of NaCl5%	Pyrazinamidase	Reduction of tellurite potassium	Urease	Runyon group	Similarity (%)	Base pair differences	Identification	
A (2, 12, 13)	Khorramabad (Lorestan)	River sediment/forest soil	7	8–14	35	R	+	+	+	−	−	+	IV	99.69	4/1285	*M. sacrum* like	PAHs/sodium sulfate
A28	Ahwaz (Khuzestan)	River sediment	7.2	18	35	R	+	+	+	−	−	+	IV	99.69	4/1285	*M. sacrum* like	PAHs/sodium sulfate
A53	Fulad Shahr (Isfahan)	Forest soil	7.4	30	30	R	−	+	+	−	−	+	IV	97.74	22/974	*M. flavescens*	PAHs/crude oil
A82	Dorud (Lorestan)	River sediment	7.2	25	30	R	−	+	+	−	−	+	IV	97.74	22/974	*M. flavescens*	PAHs/crude oil
A (113, 88)	Yazd (Yazd)	Hospital water	7.4	14	30	R	−	+	+	−	−	+	IV	97.74	22/974	*M. flavescens*	PAHs/crude oil
A38	Shazand (Markazi)	Oil refinery soil	8.5	6	35	R	+	−	+	+	−	−	IV	100	0/1074	*M. conceptionense*	Dibenzothiophene
A55	Abadan (Khuzestan)	Oil refinery soil	7.6	24	25	R	+	+	+	−	−	+	IV	99.9	1/1074	*M. monacense*	PAHs/crude oil/pristan
A75	Isfahan (Isfahan)	Porcelain wastewater	6.5	20	25	R	+	+	+	−	−	+	IV	99.9	1/1074	*M. monacense*	PAHs/crude oil/pristan
A6	Dezful (Khuzestan)	Dam water	7.6	13	30	R	−	+	+	−	+	+	IV	99.56	6/1378	*M. vanbaalenii* like	PAHs
A16	Arak (Markazi)	Salt Lake sediment	8	4	25	R	−	−	−	+	−	+	IV	98.22	24/1352	*M. gadium* like	Sodium sulfate
A4	Khorram abad (Lorestan)	Land farm soil	7	14	25	R	+	+	−	−	+	+	IV	99.48	7/1356	*M. tusciae* like	PAHs
A23	Kish (Hormozgan)	Sea sediment	7.9	20	30	R	+	−	+	−	−	+	IV	98.1	18/916	*M. pallens* like	PAHs
A7	Isfahan (Isfahan)	Hospital water	7.6	12	30	S	−	−	+	+	−	−	II	99	9/960	*M. cookii* like	–
A15	Omidie (Khuzestan)	Oil contaminated soil	6.4	25	25	R	+	+	+	−	−	+	IV	99.9	1/1023	*M. porcinum*	PAHs/crude oil/phenol
A18	Khorramabad (Lorestan)	Hospital water	7.6	10	35	S	−	−	+	+	+	−	I	100	0/999	*M simiae*	–
A5	Bushehr (Bushehr)	Sea sediment	7.2	24	30	R	+	+	+	−	−	+	IV	100	0/872	*M. fredriksbergense*	PAHs/crude oil
A9	Isfahan (Isfahan)	River sediment	7.8	8	30	R	+	+	+	−	−	+	IV	100	0/872	*M. .fredriksbergense*	PAHs/crude oil
A8	Shahin Shahr (Isfahan)	Oil refinery soil	8.3	18	30	R	+	−	+	−	−	−	IV	99.4	4/663	*M. celeriflavum*	PAHs
A14	Omidie (Khuzestan)	Oil well sediments	6.9	20	25	R	+	+	−	−	+	+	IV	99.2	11/1378	*M. vaccae* like	PAHs/crude oil
A33	Bandar anzali (Gilan)	River sediments	6.8	20	35	R	+	+	+	+	+	−	IV	99.7	3/983	*M. novocastrense*	Sulfate sodium
A (24, A25, A52)	Isfahan (Isfahan)	Hospital water and soil	7–8.8	12–28	35	S	+	−	−	−	+	−	II	99.6–99.9	1–4/995	*M. paragordonae*	–
A90	Shahr Kurd (kohkiloye)	Hospital water	7.6	14	35	S	+	−	−	−	+	−	II	99.9	1/995	*M. paragordonae*	–
A11	Dorud (Lorestan)	Cement factory soil	6.8	14	35	R	+	−	+	+	+	+	IV	100	0/1044	*M. aurum*	PAHs/PVC/morpholine/piperidin
A89	Kermanshah (Kermanshah)	Petrochemical factory soil	7	16	35	R	+	−	+	+	+	+	IV	100	0/1044	*M. aurum*	PAHs/PVC/morpholine/piperidin
A43	Hamadan (Hamadan)	Power plant soil	8.5	6	35	R	+	−	−	+	+	+	IV	99.12	11/1253	*M. neoaurum*	PAHs/crude oil/pristan
A27	Kerman (Kerman)	Sarcheshmeh copper mine soil	8.4	16	25	R	−	+	+	−	+	−	IV	99.63	4/1067	*M. obuense*	PAHs/methoxy chloro ethane
A85	Chabahar (Sistan and balochestan)	Sea sediments	7.4	14	35	R	−	+	−	+	+	−	IV	99.64	5/1385	*M. phocaicum* like	PAHs/crude oil/fluoro-glycofen ethyl
A3	Ahwaz (Khuzestan)	River sediments	7.8	16	35	R	−	+	−	+	+	−	IV	99.64	5/1385	*M. phocaicum* like	PAHs/crude oil/fluoro-glycofen ethyl
A (44, 49)	Khorramabad (Lorestan)	Forest soil	7 –7.6	14–26	35	R	−	−	−	+	−	−	IV	98.56–100	0–12/1247	*M. fortuitum*	Natural rubber/phenols/squalene
A63	Isfahan (Isfahan)	Hospital water	7.4	16	35	R	−	−	−	+	−	−	IV	100	0/1247	*M. fortuitum*	Natural rubber/phenols/squalene
A (69, 91)	Najaf abad (Isfahan)	Tile factory sewage	7.6	22	35	R	−	−	−	+	−	−	IV	98.56–100	0–12/1247	*M. fortuitum*	Natural rubber/phenols/squalene
A115	Ramsar (Mazandaran)	Forest soil	7	18	35	R	−	−	−	+	−	−	IV	98.88	6/1247	*M. fortuitum*	Natural rubber/phenols/squalene

*Opt. Tm* optimum temperature, *Similarity* % similarity to the nearest validated species, *Base pair differences* the number of nucleotide differences between the isolates and the nearest validated species, *R* resistance

The samples were processed based on standard procedures. In summary, the aquatic samples were transported at 4 °C to the laboratory and processed within a maximum period of 24 h. The collected water samples were treated with 0.005% cetylpyridinium chloride (CPC) for 15 min to reduce the number of not-desirable microbial contaminants such as fungi, Protista and other bacteria. Afterwards, the pretreated samples were subjected to vacuum filtration (cellulose nitrate 0.45 µm, Sartorius AG, Gottingen, Germany). The filters were rinsed and mashed in tubes containing 15 ml of distilled water. Almost 100 µl aliquots of dissolved samples were transferred into the tubes of Löwenstein–Jensen (LJ) and Sauton’s media (Allen 1998) and incubated at constant temperatures of 25, 30 and 35 °C in an atmosphere of 5% CO_2_.

For soil and dust, 15–30 g of samples were taken from 3 to 5 cm depth of the sampling points and transferred to the laboratory. Five grams of samples were transferred to 50 ml sterile centrifuge tubes containing 20 ml sterile distilled water and centrifuged at 4300 × *g* for 20 min at room temperature. The pellets and supernatants were decontaminated in separate tubes by addition of 3% sodium lauryl sulfate and 1% NaOH (Kamala et al. 1994). Afterwards, 100 µl of the decontaminated samples were used to inoculate into Löwenstein–Jensen (LJ) and Sauton’s media and incubated at temperatures of 25, 30 and 35 °C in an atmosphere of 5% CO_2_ for 12 weeks (Kamala et al. 1994; Lahiri et al. 2014).

For sediment samples, up to 3 g of samples were stirred for 30 min in 100 ml of sterile Ringer’s solution (5% v/v). The suspension was homogenized, tenfold serial dilutions were prepared in sterile water and 200 μl of each pretreated 10^−2^, 10^−3^, and 10^−4^ dilutions was inoculated into the Sauton’s media supplemented with antifungal and antibacterial antibiotics including kanamycin, nystatin and nalidixic acid (each at 50 µg/ml). The samples were incubated for 3 weeks at temperatures of 25, 30 and 35 °C in an atmosphere of 5% CO_2_ (Baskaran et al. 2011).

The details of environmental samples tested in the current study are given in Table 1.

### Conventional identification of the isolates

The isolates were characterized phenotypically by the use of conventional phenotypic and biochemical tests (Wayne 1984). The tests included acid-fast staining, colony characterization, the growth rate at temperatures of 25, 30 and 35 °C, and the standard biochemical assays, i.e., semi quantitative and heat-stable (68 °C) catalase production, tween opacity, pigment production, urease and pyrazinamidase activity, nitrate and tellurite reduction, and niacin accumulation.

### Molecular identification of the isolates

#### DNA extraction and purification

Chromosomal DNA was extracted using simple boiling method or the modified Pitcher method (Pitcher et al. 1989; Rahdar et al. 2017; Shojaei et al. 2011). In brief, the simple boiling method was performed as follows: a few colonies of bacteria added into 2 ml of TE buffer (tris EDTA), and boiled for 30 min, centrifuged at 11,900×*g* for 10 min. The supernatant was transferred to a sterile microtube and centrifuged at 20,000×*g* for 10 min. The modified Pitcher method included the lysis of actinomycetes cells obtained with the pretreatment with lipase (2 mg/ml) followed by the cell wall disruption using lysozyme (200 mg/ml final concentration) and proteinase K (300 μg/ml final concentration) in the presence of 3% sodium dodecyl sulfate (SDS). The DNA-containing aqueous phase was purified with phenol–chloroform–isoamyl alcohol (25:24:1, vol/vol/vol) and chloroform-isoamyl alcohol (24:1, vol/vol). The precipitation of DNA by either of two methods, was carried out using ammonium acetate (7.5 mol/l) and ethanol at −20 °C. The Precipitated DNA was washed with 70% ethanol and re-suspended in 100 µl of Milli-Q water.

### Molecular identification of mycobacterial isolates

The environmental isolates that were identified phenotypically as *Mycobacterium* were further verified to the genus level using a specific PCR protocol based on a 228-bp fragment of the 65-kDa heat shock protein (*hsp*65). The primers included *hsp*65F: 5′**-**CTGGTCAAGGAAGGTCTGCG-3′, and *hsp*65R: 5′**-**GATGACACCCTCGTTGCCAAC-3′) as recommended by Khan and Yadav (2004). For identification of the isolates at the species level the amplification and direct sequence analysis of 16S rRNA gene was used as described previously (Shojaei et al. 2011) using the primers 27F: 5′**-**AGA GTT TGA TCM TGG CTC AG-3′and 1492R: 5′**-**CG GTT ACC TTG TTA CGA CTT-3′) (Wilson et al. 1990). The sequencing was performed by ABI 3100 genetic analyzer in Bioneer Company (South Korea). The obtained sequences were aligned manually with all existing sequences of the closely related mycobacteria retrieved from the GenBankTM database, compared with the relevant sequences and analyzed using the jPhydit program (Jeon et al. 2005).

### Nucleotide sequence accession numbers

The GenBank accession numbers for the 16S rRNA sequencing of the mycobacteria isolated in this study are; *M. coockii* like (JX566888)*, M. simiae* (KF028776), *M. frederiksbergense* (KF019696), *M. sacrum* like (KU564076)*, M. vanbaalenii* like (KU564074)*, M. gadium* like (KU564078)*, M. aurum* (KU564075)*, M. tusciae* like (KU564073), *M. novocastrense* (NR_029208), *M. obuense* (KF028777) and *M. phocaicum* like (KF019699).

### Strain culture collection numbers

The culture collection numbers for two potentially novel strains of mycobacteria in this study are listed below: *M. cookii* like (A7) JCM 30922 and CCUG 67561 and *M. phocaicum* like (A3 and A85) JCM 30989 and CCUG 67787.

### Bioremediation analysis

The biodegradation activity of the strains isolated from environmental samples in the current study was evaluated according to Kanaly and Harayama (2000). The details are as follows:

### Chemicals and media

PAHs mix solution (1-1) [Acenaphthene, Acenaphthylene, Anthracene, Benzo(b)fluoranthene, 1,2-Benz anthracene, Benzo(a)pyrene, Benzo(k)fluoranthene, Benzo(g,h,i)perylene, Chrysene, Dibenz(a,h)anthracene, Fluoranthene, Fluorene, Indeno(1,2,3-cd)pyrene, Phenanthrene, Naphthalene, Pyrene] were purchased from AccuStandard. PAHs stock solution was 0.2 mg/ml in dichloromethane and methanol. Phenol was purchased from Merck, Germany. Phenol stock solution was 10 mg/ml in deionized water. Sodium sulfate was purchased from Merck, Germany. Sodium sulfate stock solution was 10 mg/ml dissolved in deionized water. All other solvents and chemicals used were of reagent grade. Mineral Salt Medium (MSM) contained (g/l), g/l were KH_2_PO_4_ (0.42), K_2_HPO_4_ (0.375), (NH_4_)_2_SO_4_ (0.244), NaCl (0.015), CaCl_2_·2H_2_O (0.015), MgSO_4_·7H_2_O (0.05), and FeCl_3_·6H_2_O (0.054).

Evaluation of biodegradation activity of isolates was carried out by adding 1 ml of direct colony suspension is made in normal saline and turbidity adjusted to 0.5 McFarland standard (1.5 × 10^8^ CFU/ml) into 100 ml aliquots of % 1 PAHs, % 1 phenol and % 1 sodium sulfate enriched MSM were prepared in 250 ml flask and incubated for 6 days at 30 °C in an orbital shaker (90 rpm). To evaluate the bacterial growth, samples were collected at 24-h intervals and the absorbance at 560 nm was measured by Spectrophotometer.

Showing sign of growth in the media indicates consumption and/or decomposition of material by studied isolates. Standard procedure was used to final confirmation of degradation of the studied material, (Chesnin and Yien 1951; Gibbs 1927; Manoli and Samara 1996) explained in the following method.

### Determination of PAHs degradation

An amount 5 ml of the MSM medium containing PAHs that showed bacterial growth was transferred to a screw cap glass tube and supplemented with 0.6 ml of a mixture of tetrachlorethylene and methanol (1:100) as the extraction solvent, vortexed for 10 s, then centrifuged at 3000×*g* for 10 min. The organic phase was then collected and transferred to a clean tube to be further analyzed by HPLC. PAHs were analyzed by Manager 5000 HPLC systems (Knauer, Germany). A reversed phase column C18 (60 × 2 mm, particle size 2.2 µm) using mobile phase with acetonitrile/water (gradient elution 20 min at a constant flow rate 5:95, and 0.3 ml/min 50 °C) was used to separate PAHs (Manoli and Samara 1996). The UV absorbance at 254 nm was measured and the PAHs content of the sample was calculated using the standard curve and function calculated using sterile PAHs standards.

### Determination of phenol degradation

An amount of 5 ml of MSM medium containing phenol which showed bacterial growth was transferred to a sterile tube and the pH was adjusted to 8.0, then subjected to centrifugation at 2700*g* for 20 min. An amount of 150 µl of the collected supernatant was mixed with 30 µl NaHCO_3_, and 20 µl of Gibbs reagent (2,6-dichloroquinone 4-chloroimide) was added to the mixture and shacked for 30 min at room temperature (Gibbs 1927). The UV absorbance at 620 nm was recorded and the phenol content determined using a standard curve and function calculated using sterile phenol standards.

### Determination of sodium sulfate degradation

An amount of 5 ml of MSM medium containing sodium sulfate was transferred to a sterile tube. One ml 1% acetic acid and 1 ml acetate buffer was added and mixed for 3 min. Afterwards, 1 ml of barium chloride was added and mixed for another 3 min. The turbidity was calculated using Spectrophotometry at a wavelength of 420 nm (Chesnin and Yien 1951). The amount of consumed sodium sulfate was measured by using the standard curve calculated using sterile sodium sulfate standards.

## Results

### Isolation and characterization of strains

The recorded temperature and pH of the soil samples were in the range of 4–30 °C and 6.0–8.0 respectively. For wastewater and sediment samples, these measures were in the range of 7–24 °C and 6.8–7.8, respectively. The corresponding figures for the water samples were 5–23 °C and 7.0–8.2, respectively, and the total dissolved solids (TDS) for the water samples ranged between 600 and 1200 mg/l (Table 1).

The details of water, soil, wastewater and sediment samples and the isolates key properties are presented in Table 1.

From 90 water, soil, wastewater and sediment samples, a total number of 39 isolates were recovered and identified as non-tuberculosis mycobacteria based on culture, morphological and biochemical features, and by molecular analysis.

The 16S rRNA gene sequencing of the isolates revealed that all isolates had nucleotide signatures of mycobacteria at positions 70–98 (A–T), 293–304 (G–T), 307 (C), 328 (T), 614–626), 661–744 (G–C), (A–T), 631 (G), 824–876 (T–A), 825–875 (A–T), 843 (C), and 1122–1151 (A–T) (Stackebrandt et al. 1997). Of these 39 isolates, 15 were slowly growing mycobacteria and 24 were rapidly growing mycobacteria. All rapidly growing isolates contained the short helix 18 (position 451–482) that is characteristic of the rapidly growing mycobacteria. In contrast, all slowly growing isolates, except for the isolate A18, characterized by an extended helix, which is present in slowly growing mycobacteria. The isolate A18 was an exception in this scheme since it belonged to *M. simiae* complex that contrary to other slow growing mycobacteria present the signature of rapid growers (short helix 18) in the 16S rRNA (Tortoli 2003).

Based on morphological, culture and biochemical properties and the genus specific marker, i.e., the presence of a 228-bp fragment of the *hsp*65, all 39 isolates were identified as *Mycobacterium* of which, 2 isolates fit into Runyon group I, 11 isolates in Runyon group II, 2 isolates in Runyon group III and 24 isolates, i.e., the rapidly growing mycobacteria were classified in Runyon group IV (Table 1). The isolates belonged to 14 validated species and 7 unknown or potentially novel species. The most prevalent mycobacteria were *M. fortuitum* (6 isolates, 15.38%). This followed by *M. flavescens* and *M. paragordonae* (4 isolates each; 10.25%) and a potentially novel species closely related to uncharacterized species *M. sacrum* (4 isolates, 10.25%). The other thirteen isolates comprised a single isolates each belonged to seven established species that were *M. conceptionense, M. porcinum, M simiae*, *M. celeriflavum, M. obuense, M. novocastrense* and *M. neoaurum,* as well as six unknown isolates potentially novel species of mycobacteria that were closely related to *M. tusciae* (isolate A4), *M. vanbaalenii* (isolate A6), *M. gadium* (isolate A16), *M. pallens* (isolate A23), *M. cookii* (isolate A7), and *M. vaccae* (isolate A14).

**Fig. 2:**
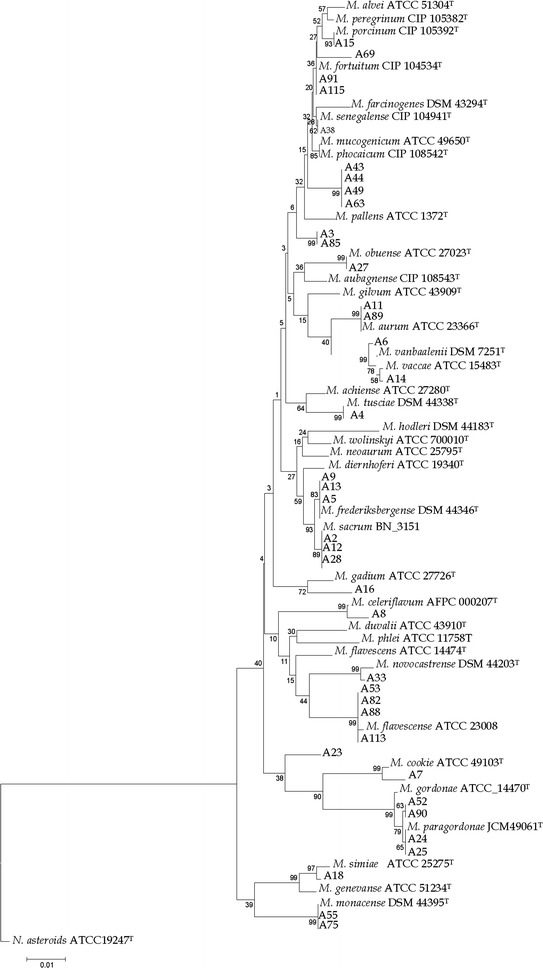
16S rRNA sequence based phylogenetic tree for Iranian biodegrading NTM isolates and the nearest validated species of mycobacteria by using the neighbor-joining method. The figures at each node represent bootstrapping values. The tree was rooted with *N. asteroides*

The relationship between our isolates and the standard established species of mycobacteria was supported by a high bootstrap value in phylogenetic tree based on 16S rRNA gene depicted by MEGA 6. The corresponding value for the isolates that showed substantial nucleotide differences with the known mycobacterial species and named as novel species requiring further investigation found to be rather low (Fig. 2).

### Bioremediation analysis

Based on biodegradation activity, our isolates can be classified in three categories:

1. The isolates without biodegradation activity that included, four isolates of *M. paragordonae*, i.e., the isolates A24, A25, A52 and A90 and one isolate of *M. simiae* that is, the isolate A18 (Table 1).
2. The isolates with biodegradation activity based on the previous reports by other investigators, that included two isolates of *M. frederiksbergense*, i.e., A5 and A9, with capability of PAHs degradation (Willumsen et al. 2001), two isolates of *M. phocaicum,* i.e., A3 and A85 with capability of PAHs and fluoroglycofen ethyl degradation (Chen et al. 2011), one isolate *M. obuense,* i.e., A27 with capability of Methoxychlor ethane degradation (Satsuma and Masuda 2012), Two isolates of *M. aurum,* i.e., A11 and A89 with capability of PAHs, polyvinyl chloride (PVC), morpholine, thiomorpholine and piperidin degradation (Combourieu et al. 1998; Hartmans and De Bont 1992), One isolate of *M. conceptionense,* i.e., A38 with capability of dibenzothiophene degradation (Akhtar et al. 2016), One isolate of *M. neoaurum,* i.e., A43 with capability of pristan, soybean phytosterols, crude oil and PAHs degradation (Bastiaens et al. 2000; Mikolasch et al. 2009; Wei et al. 2010), Six isolates of *M. fortuitum,* i.e., A44, A49, A63, A69, 91 and A115 with capability of poly chlorinated and brominated biphenyls (PCB and PBBs), natural rubber and squalene degradation (Berekaa and Steinbüchel 2000; Rose and Steinbüchel 2005; Uotila et al. 1992), four isolates of *M. flavescens,* i.e., A53, A83, A88 and A113 with capability of crude oil and PAHs degradation (Miller et al. 2004), and two isolates of *M. monacense*, i.e., A55 and A75 with capability of crude oil and PAH degradation (Miller et al. 2004).
3. The isolates that were either the previously established mycobacteria without known biodegradation activity or the novel species that were first isolated and reported in the current study as capable of biodegradation of environmental chemical pollutants.

**Fig. 3:**
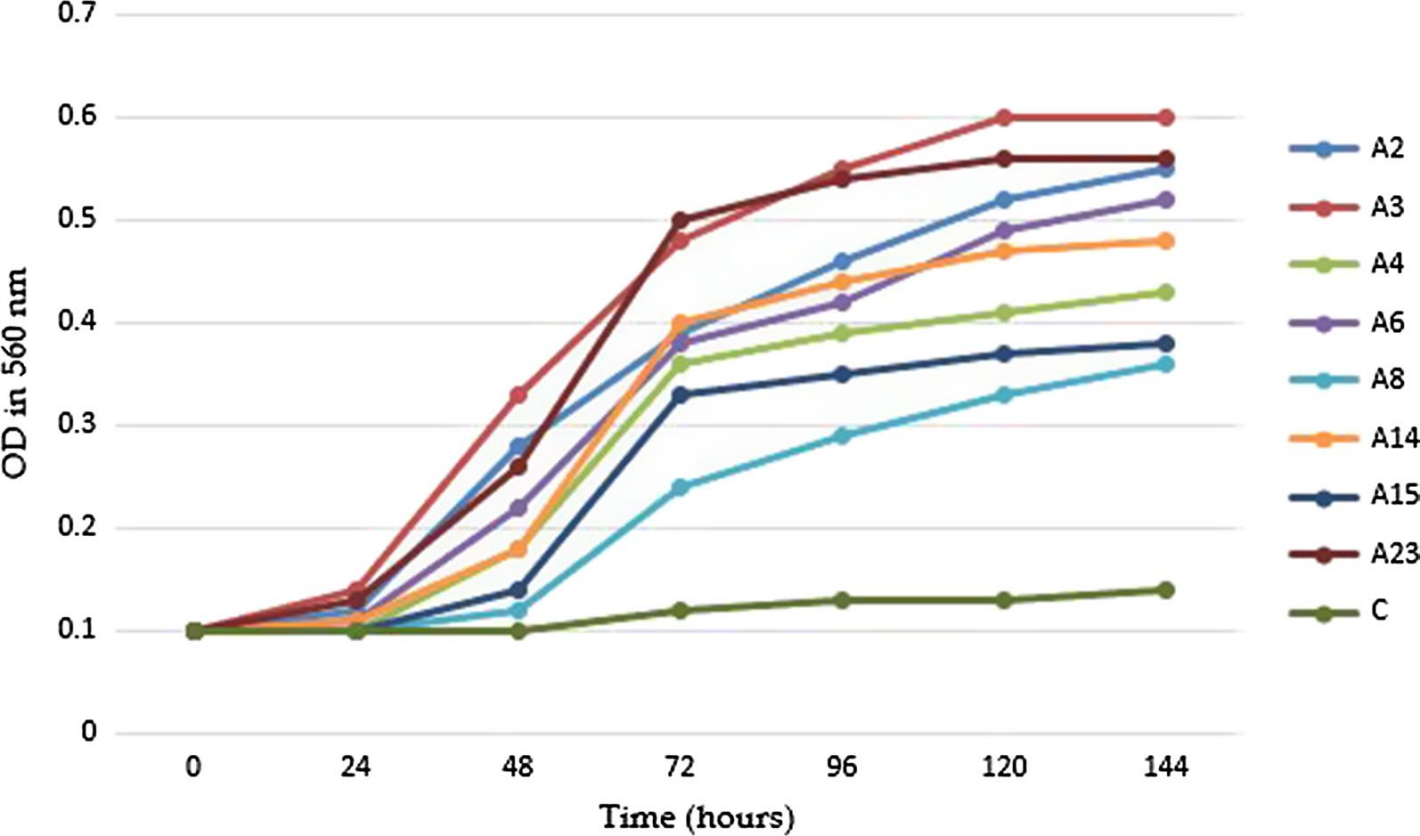
Growth curves of Iranian isolates of mycobacteria over a 24 h. Incubation period at 30 °C in the presence of PAHs

A growth curve was plotted with O.D at 560 nm on Y axis and time in hours on axis respectively. On the basis of growth curve plotted, it was found that isolates A3 and A85 were identified as *M. phocaicum* like had maximum growth against a concentration of 0.2 µg/ml for PAHs mix solution, followed by the isolates A2, A12, A13 and A28 were identified *as M. sacrum*-like, the isolate A4 was identified *as M. tusciae* like, the isolate A6 was identified *as M. vanbaalenii* like, the isolate A8 was identified *as M. celeriflavum,* the isolate A14 was identified *as M. vaccae* like, the isolate A15 was identified as *M. porcinum* and the isolate A23 was identified as *M. pallens* like. The growth curves are shown in Fig. 3. A3 and A85 was found to be highest growth rate in presence of PAHs followed by the growth of isolate A23, A2, A6 and A14 isolate and the isolate A8 was found to lowest growth rate. The growth response indicates that, organisms were able to utilize PAHs mix solution as their sole carbon source. As the growth proceeded, the metabolites produced during the degradation of PAHs were toxic at a higher level which reduced the growth rate (Table 1; Fig. 3).

**Fig. 4:**
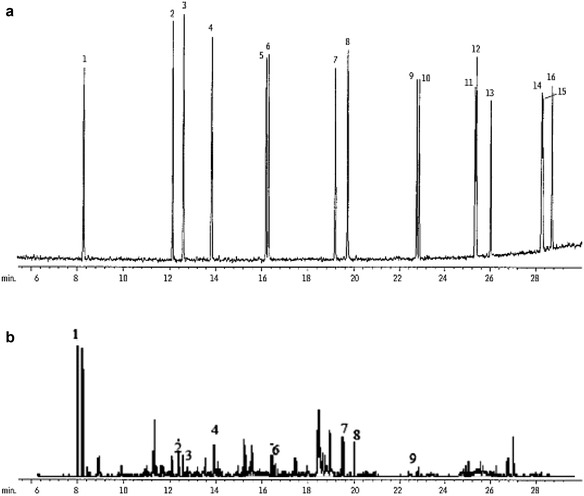
HPLC chromatograms of PAHs mix solution by selected mycobacterial isolates, **a** control samples, **b** after 144 incubation at 30 °C. (1) Naphthalene, (2) acenaphthylene, (3) acenaphthene, (4) fluorene, (5) phenanthrene, (6) anthracene, (7) fluoranthene, (8) pyrene, (9) benzo[a] anthracene, (10) chrysene, (11) benzo[b]fluoranthene, (12) benzo[k]fluoranthene, (13) benzo[a]pyrene, (14) indeno[1,2,3-cd]pyrene, (15) dibenzo[a,h]anthracene

According to the chromatogram obtained from the analysis, the results are as follows. Isolate A3 and A83 showed a higher rate of mixed PAHs degradation with 90% degradation of it (Fig. 4).

**Fig. 5:**
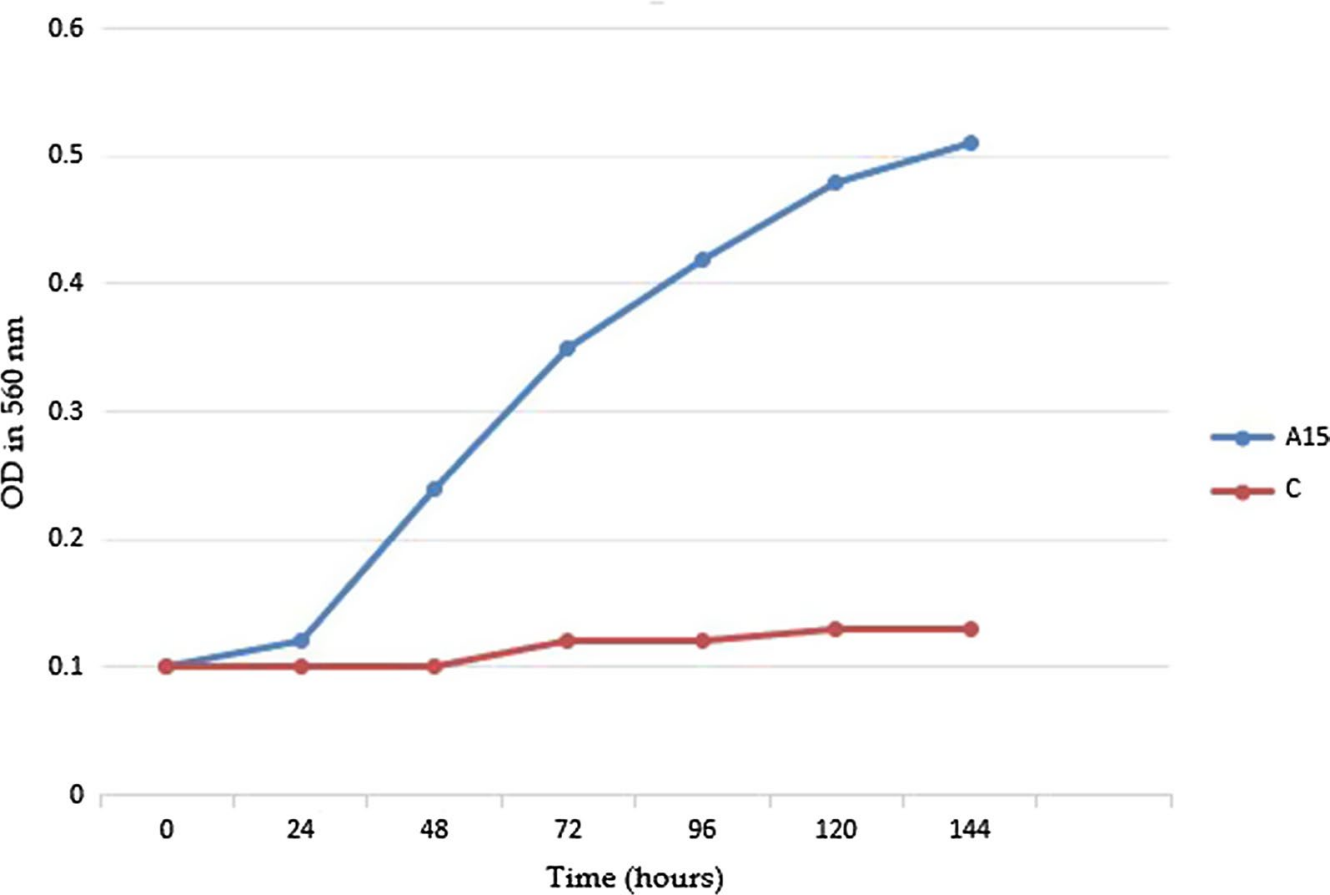
Growth curves of Iranian *mycobacterium* isolates over a 24 h. Incubation period at 30 °C in the presence of phenol

On the basis of growth curve plotted, it was found that isolate A15 was identified as *M. porcinum* had maximum growth against a concentration of 1 mg/ml for phenol. The growth response indicates that, organisms were able to utilize phenol as their sole carbon source (Table 1; Fig. 5). The results of Gibbs method showed that isolate A15 was able to degrade the 87% of phenol after 144 h.

**Fig. 6:**
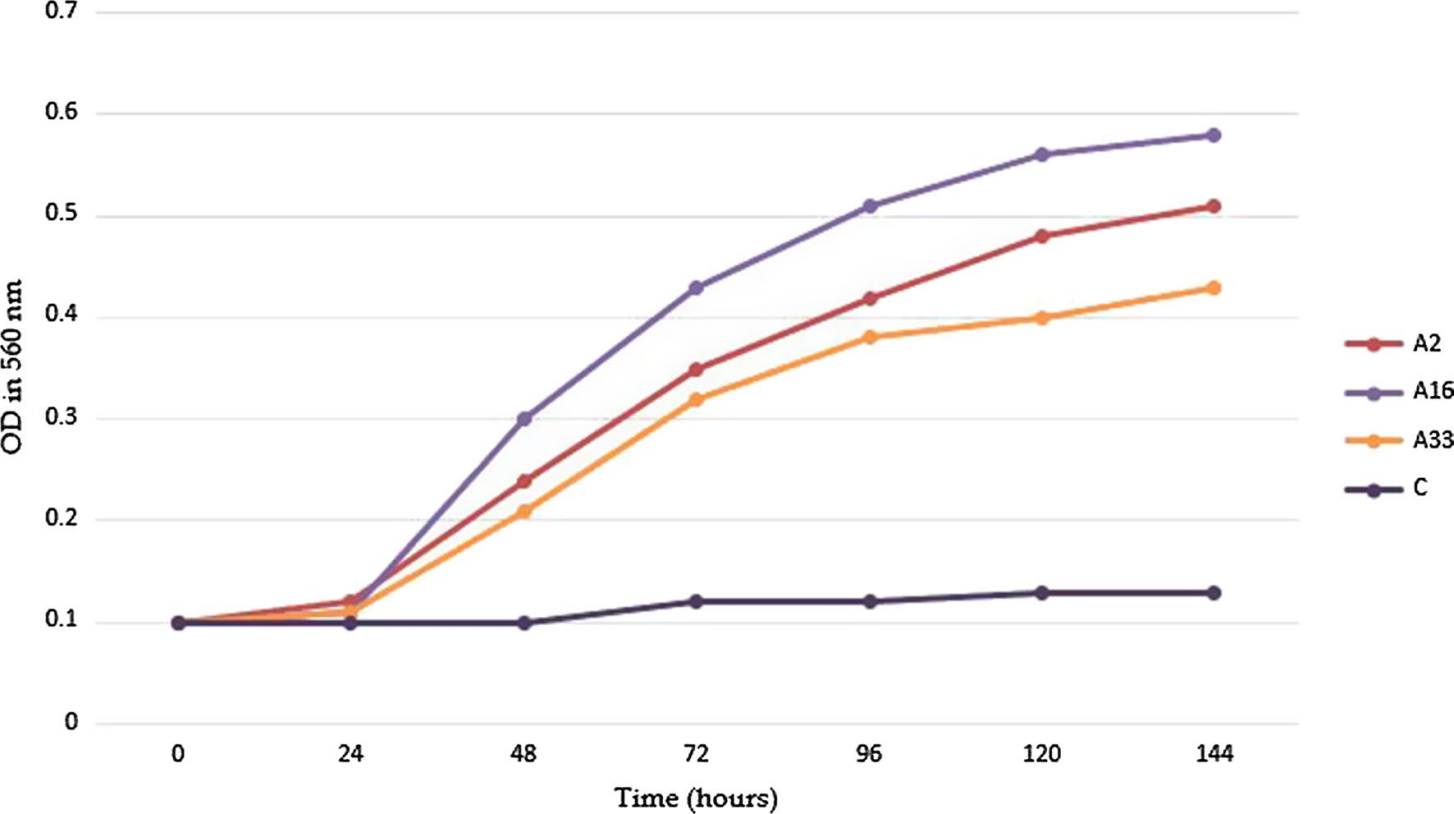
Growth curves of Iranian isolates of mycobacteria over a 24 h. Incubation period at 30 °C in the presence of sodium sulfate

On the basis of growth curve plotted, it was found that isolate A16 had maximum growth against a concentration of 1 mg/ml for sodium sulfate, followed by the isolates A2, A12, A13, A28 and the isolate A33 was identified as *M. novocastrense*. The growth curves are shown in Fig. 6 isolate A16 was found to be highest growth rate in presence of %1 sodium sulfate, followed by the growth of isolate A2 and isolate A33 and the other tested isolate was not able to grow significantly (Table 1; Fig. 6). The growth response indicates that, organisms were able to utilize sodium sulfate as their sole carbon source.

The results of turbidimetry method, indicates that isolate A16 showed highest sodium sulfate biodegradation activity and was able to degrade 100% of the sodium sulfate followed by A2 and A33 that was able to degrade 80 and 65% of sodium sulfate in medium after 144 h respectively.

## Discussion

Since the exploration of oil in 1908, Iran has experienced an economic transition, i.e. transformed from traditional agriculture led to industry led. Development, and industrialization in particular, have made immense positive contributions to health, including greater personal and social wealth, as well as vastly improved health, transportation and communication. Iranian people are living longer and are healthier than they were centuries and even decades ago. However, industrialization has also had adverse health consequences not only for workforces, but for the general population as well. These effects have been caused either directly by exposure to safety hazards and harmful agents, or indirectly through environmental degradation locally and regionally. Iranian people are being exposed to these environmental health hazards through a range of ways that include traditional hazards of industrial contamination of air, water, food and land, as well as new pollution phenomena such as dust and sand storms.

Destroyed ecosystems and health risks have emphasized on the necessity for cleanup and remedies for soil and water, which have prompted many researchers to develop environment clean up strategies. These approaches include the physical, chemical and thermal processes (Arias-Estévez et al. 2008; Schaer et al. 2001). These techniques however have some adverse effects on the environment and are also expensive (Mulligan et al. 2001; Virkutyte et al. 2002). Recently, biological techniques like phytoremediation and bioremediation are being evaluated for the remediation of environment contaminated with chemical pollutants (Adams et al. 2015; Brown et al. 2011; Susarla et al. 2002).

Bioremediation is a waste management technique that involves the use of naturally occurring organisms to neutralize pollutants from a contaminated site. Up to now, many bacterial strains, such as *Pseudomonas, Alcanivorax, Acinetobacter, Rhodococcus,* and *Bacillus* have been isolated from soil and water, and were effectively used for the bioremediation of petroleum contaminants (Das and Mukherjee 2007; Girma 2015; Ron and Rosenberg 2002). Depending on the site and its contaminants, bioremediation may be safer and less expensive than alternative solutions such as incineration or landfilling of the contaminated materials. It also has the advantage of treating the contamination in place so that large quantities of soil, sediment or water do not have to be dug up or pumped out of the ground for treatment (Adams et al. 2015).

Till date several investigators have described the ability of sludge-derived mycobacteria to degrade chemical pollutants (Guo et al. 2010; Miserez et al. 1999). Mycobacteria have shown ability to degrade polychlorophenols, heavy metals and diverse PAHs (Girma 2015; Kumar et al. 2011; Xun 2012).

This study was undertaken to screen various Iranian ecosystems in search of mycobacteria with capability to degrade environmental contamination and in particular the oil contaminating pollutants.

A total of 39 biodegrading mycobacteria were isolated from 90 samples collected from the diverse ecosystems of Iran. The isolates A5 and A9 were identified as *M. frederiksbergense.* This organism is a rapidly growing scotochromogenic *Mycobacterium* which was first isolated and characterized in 2001 from soil in Denmark (Willumsen et al. 2001). *M. frederiksbergense* is able to degrade light and heavy chain PAHs (Willumsen et al. 2001).

The isolates A3 and A85 were identified as *M. phocaicum.* This organism was first isolated and characterized in 2006 from clinical samples (Adékambi et al. 2006a). Afterwards, Chen and his colleagues found out that *M. phocaicum* was able to degrade fluoroglycofen ethyl and PAHs (Chen et al. 2011).

The isolate A27 was identified as *M. obuense*. This organism was first isolated and characterized in 2006 from clinical samples (Tsukamura and Mizuno 1971). In subsequent studies it was reported that this *Mycobacterium* has the capacity to degrade PAHs and Methoxychlor ethane which is an organochlorine insecticide (Satsuma and Masuda 2012).

The isolates A11 and A89 were identified as *M. aurum* that was first isolated and characterized in 1993 from environmental samples (Yokota et al. 1993). Based on the results of the current study and the studies by other investigators, *M. aurum* has the ability to degrade polyvinyl chloride, PAHs, morpholine, thiomorpholine and piperidin, which is a common additive for pH adjustment in both fossil fuel and nuclear power plant steam systems (Combourieu et al. 1998; Hartmans and De Bont 1992).

The isolate A33 was identified as *M. novocastrense* which was first isolated and characterized from clinical samples (Shojaei et al. 1997), However there has been no reports on its biodegradation activity. In the present study, we showed that *M. novocastrense* has the capacity to degrade sodium sulfate.

The isolate A38 was identified as *M. conceptionense*. This organism is a rapidly growing scotochromogenic *Mycobacterium* which was first isolated and characterized in 2006 from clinical samples (Adékambi et al. 2006b). It has been shown that *M. conceptionense* is able to desulfurize dibenzothiophene which is an organosulfur compound used in oil refinery (Akhtar et al. 2016).

The isolate A43 was identified as *M. neoaurum* which was first isolated and characterized in 1972 from clinical samples (Tsukamura and Mizuno 1972). In several studies the capability of *M. neoaurum* to degrade PAHs, crude oil and pristan (used as a lubricant, a transformer oil, an immunologic adjuvant, and an anti-corrosion agent) was reported (Bastiaens et al. 2000; Mikolasch et al. 2009; Wei et al. 2010).

The isolates A44, A49, A63, A69, A91 and A115 were identified as *M. fortuitum*. This species has shown an extended capacity for degradation of hexamethyltetracosane, halogenated phenol derivatives such as polychlorinated biphenyl (PCB) and polybrominated biphenyl (PBB) as well as natural and synthetic rubber (Berekaa and Steinbüchel 2000; Rose and Steinbüchel 2005; Uotila et al. 1992).

The isolates A53, A83, A88 and A113 were identified as *M. flavescens*. The organism was first isolated and characterized from a drug treated tuberculous guinea pig (Bojalil et al. 1962). Subsequently, it was reported that this species has ability to use and degrade PAHs and crude oil as sole carbon and energy sources (Miller et al. 2004).

The isolates A55 and A75 were identified as *M. monacense* which was first isolated from clinical samples (Reischl et al. 2006). Miller and his colleagues reported that *M. monacense* is capable of crude oil and various PAHs degradation (Miller et al. 2004).

The isolate A8 was identified as *M. celeriflavum*. This species is a rapid growing scotochromogenic *Mycobacterium* that was first isolated and characterized from clinical sample in Iran in 2015 (Shahraki et al. 2015). We isolated this organism from the river sediments and showed that it has the ability of PAHs degradation.

The isolate A15 was identified as *M. porcinum* which was first isolated from the lymph node of porcine (Tsukamura et al. 1983). We showed that this organism has the capacity of PAHs and phenol degradation.

In our study, ten isolates including A2, A4, A6, A7, A12, A13, A14, A16, A23 and A28, were found to have phenotypic and molecular characteristics of novel *Mycobacterium* species. The isolates were evaluated for biodegradation activity against PAHs, phenol and sodium sulfate. All isolates, except for the isolate A7, showed biodegradation activity. The isolates A2, A12, A13 and A28 were found to degrade PAHs and sodium sulfate. The isolates A4, A6, A14 and A23 were found to degrade the PAHs and the isolate A16 was able to degrade sodium sulfate.

The characterization of these unknown mycobacteria remains to be completed using a thorough phenotypic and molecular assays including cell wall composition analysis, sequence analysis of key genetic markers such as *gyr*B, *sec*A1, and DNA-DNA relatedness.

In conclusion, the results of our study showed that the diversity of mycobacteria, particularly prokaryotes, offers the potential for adaptation to various habitats, including environments severely contaminated with hydrocarbons and heavy metals. This broad adaptability has great value for the bioremediation of damaged ecosystems. Our study also confirms the idea that despite being abundant in environment, mycobacteria have been simply ignored for such significant usage. Indeed, there is an untapped potential with regard to bioremedial actinomycetes particularly mycobacteria that has yet to be discovered and administered in bioremediation of hazardous chemicals.

## Abbreviations

NTM: nontuberculous mycobacteria
CPC: cetylpyridinium chloride
RCF: relative centrifugal force
LJ: Löwenstein–Jensen
SDS: sodium dodecyl sulfate
TE: tris EDTA
*hsp*: heat shock protein
PAHs: polycyclic aromatic hydrocarbon
MSM: mineral salt medium
TDS: total dissolved solids
PVC: polyvinyl chloride
PCB: polychlorinated biphenyl
PBB: polybrominated biphenyl
